# Supplementary material for: Autoantibody Formation and Mapping of Immunogenic Epitopes against Cold-Shock-Protein YB-1 in Cancer Patients and Healthy Controls
Source: Cancers (Basel). 2020 Nov 25;12(12):3507. doi: 10.3390/cancers12123507 (PMC7759818; doi:10.3390/cancers12123507)
Supplement: Supplementary file 1 [file cancers-12-03507-s001.pdf]

Supplementary Materials

Autoantibody Formation and Mapping of Immunogenic Epitopes against Cold-Shock-Protein YB-1 in Cancer Patients and Healthy Controls

Ronnie Morgenroth [1], Charlotte Reichardt [1], Johannes Steffen [1], Stefan Busse [2], Ronald Frank [3], Harald Heidecke [4] and Peter R. Mertens [1,*]

[1] Clinic of Nephrology and Hypertension, Diabetes and Endocrinology, Otto-von-Guericke University Magdeburg, Leipziger Str. 40, 39120 Magdeburg, Germany; ronnie.morgenroth@med.ovgu.de; charlotte.reichardt@med.ovgu.de; johannes.steffen@med.ovgu.de

[2] Clinic of Psychiatry and Psychotherapy, Otto-von-Guericke University Magdeburg, Leipziger Str. 40, 39120 Magdeburg, Germany; Stefan.busse@med.ovgu.de

[3] AIMS Scientific Products GmbH, Galenusstr. 60, Berlin, Germany; ronald.frank@eu-openscreen.eu

[4] CellTrend GmbH, im Biotechnologiepark 3, Luckenwalde, Germany; heidecke@celltrend.de

* Correspondence: peter.mertens@med.ovgu.de; +49-391-6713236 (P.R.M.)

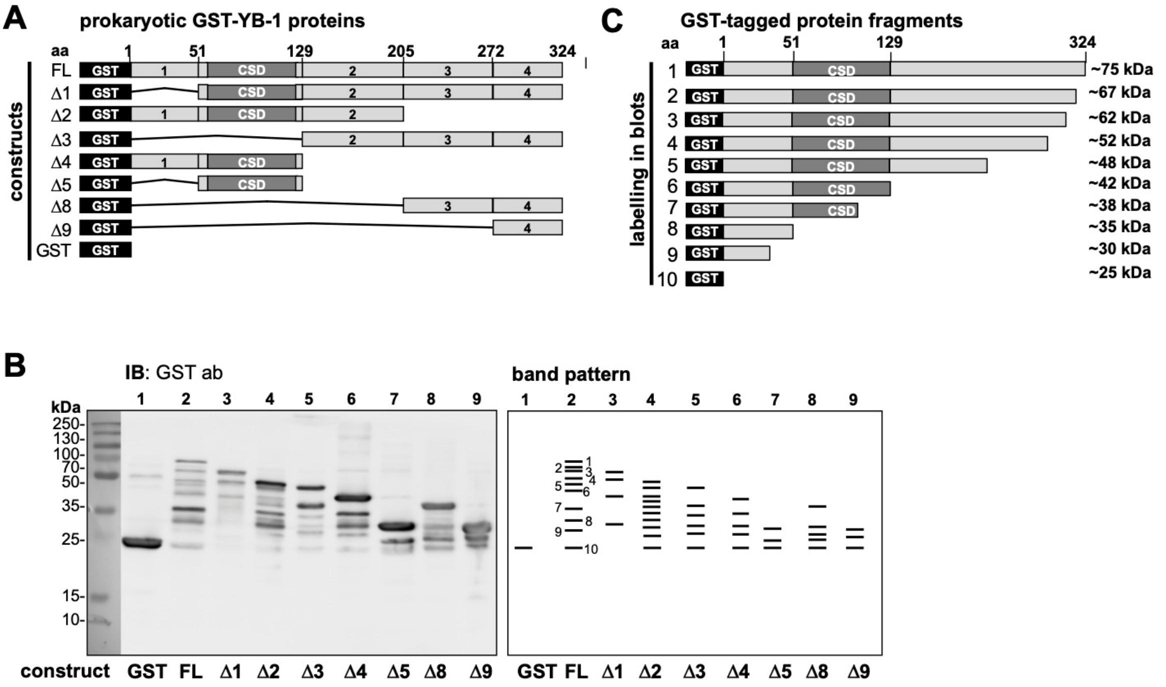

Figure S1. Characterization of spontaneous degradation/cleavage of recombinant YB-1 protein and deletion constructs

A. Scheme of GST-YB-1 protein derivatives.

B. GST-YB-1 protein constructs and GST-tag were detected by anti-GST antibody. Scheme of visualized bands with labeling of GST-YB-1 full length (FL) fragments (Lane 1). Also highlighted is GST-YB-1 FL blot with labeling of fragments (lower right corner).

C. Presentation of YB-1 fragments (10) based on abbreviated GST-YB-1 protein constructs.

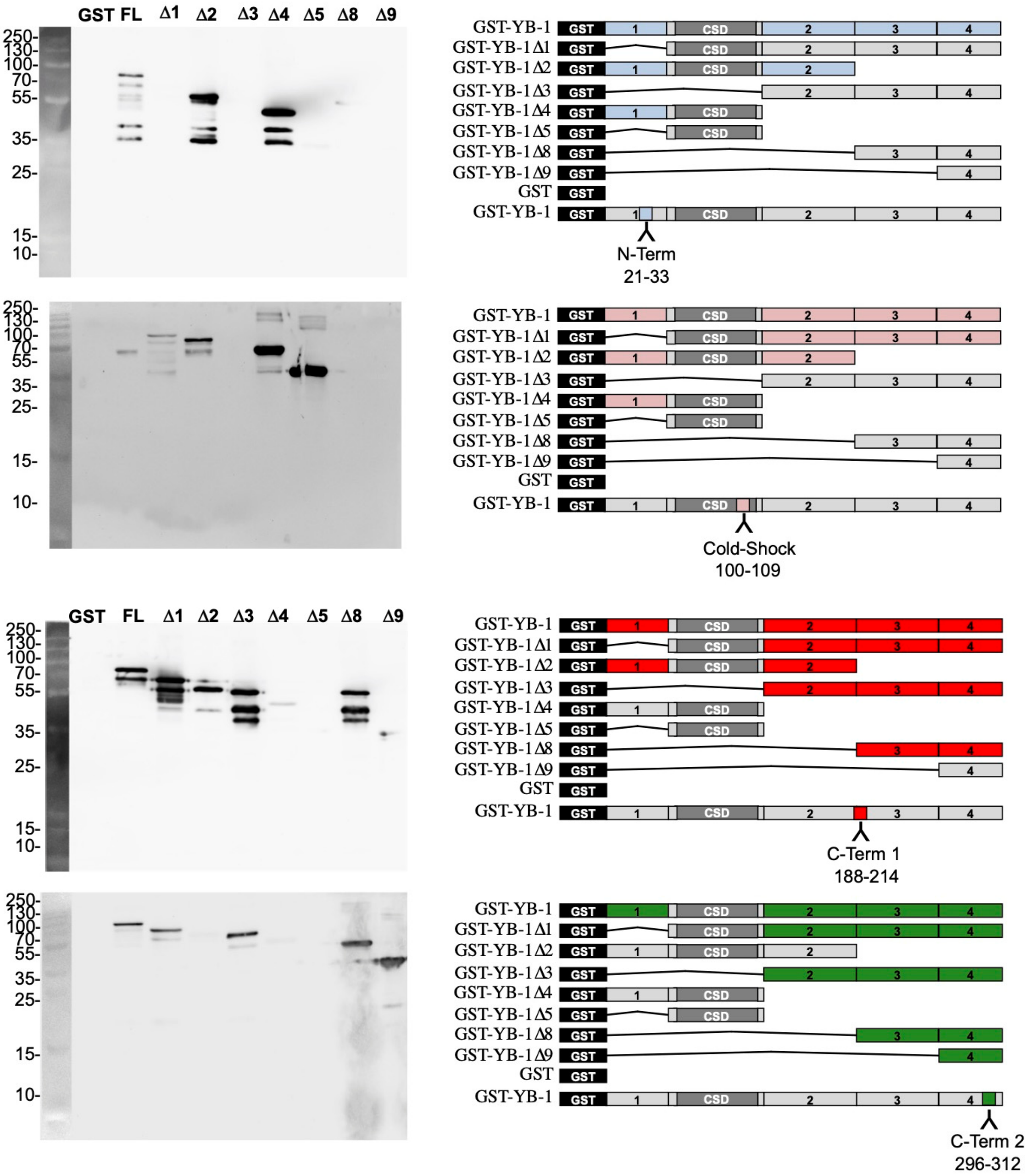

Figure S2. Epitope specificity of used YB-1 antibodies using GST-YB-1 abbreviated protein constructs.

GST-YB-1 protein and fragments were probed with peptide directed polyclonal YB-1 antisera. These YB-1 antibodies are only able to visualize those fragments that contain their epitope sequences, which are highlighted next to that.

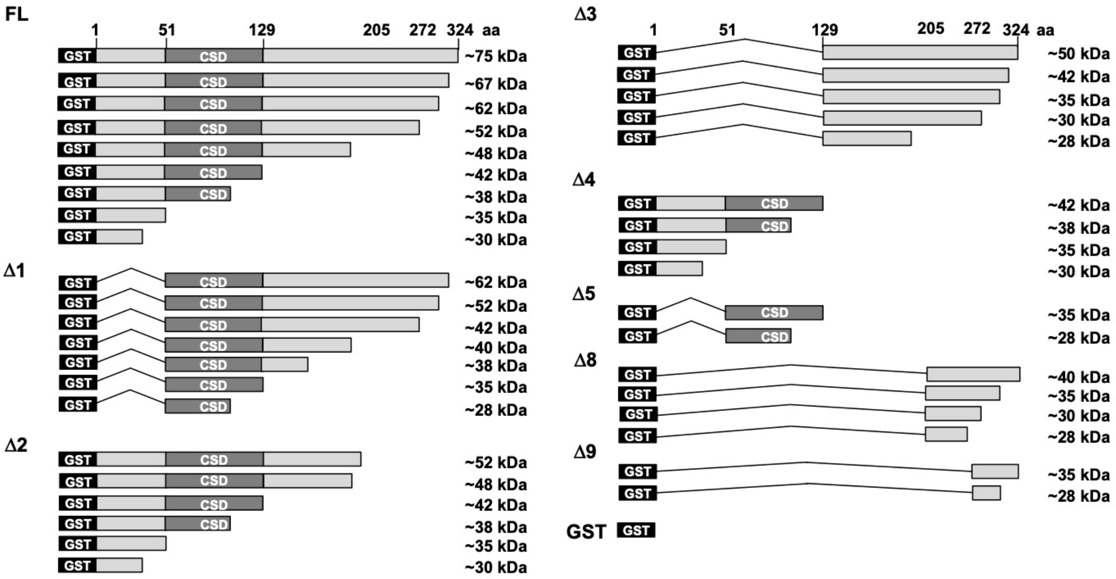

Figure S3. Potential GST-YB-1 fusion protein fragmentation.
Potential YB-1 protein fragments based on abbreviated GST-YB-1 blots are shown.

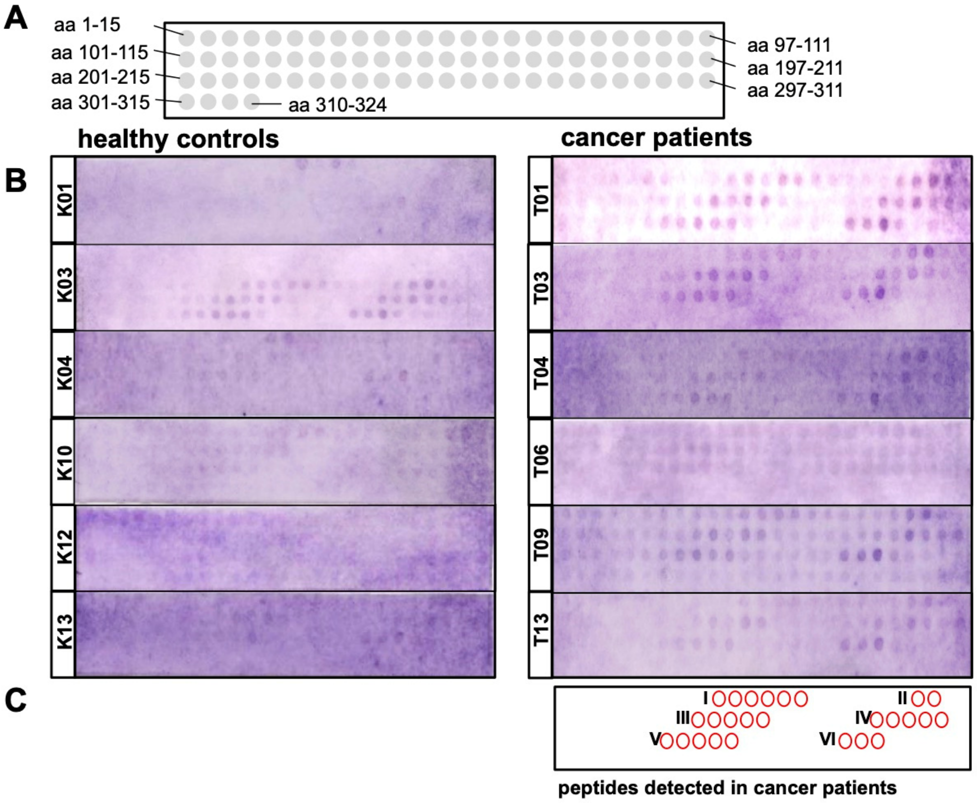

Figure S4. Peptide Array of healthy volunteers and cancer patients.

A. Lay-out of the peptide array with accompanied amino acid sequence positions.

B. Healthy controls (left panel) mostly present weak epitope binding. Cancer patients (right panel) demonstrate 6 common linear epitopes which are highlighted above.

C. Scheme of common cancer epitopes I: 41-75 II aa: 85-103; III aa: 133-163; IV: 177-207; V aa: 225-255; VI aa: 269-291.

1;1-15	MSSE AETQQPPAAPP	28;109-123	VEFD VVEGEKGAEAA	55;217-231	VMEG ADNQGAGEQGR
2;5-19	AETQ QPPAAPPAAPA	29;113-127	VVEG EKGAEAANVTG	56;221-235	ADNQ GAGEQGRPVRQ
3;9-23	QPPA APPAAPALSAA	30;117-131	EKGA EAANVTGPGGV	57;225-239	GAGE QGRPVRQNMYR
4;13-27	APPA APALSAADTKP	31;121-135	EAAN VTGPGGVPVQG	58;229-243	QGRP VRQNMYRGYRP
5;17-31	APAL SAADTKPGTTG	32;125-139	VTGP GGVPVQGSKYA	59;233-247	VRQN MYRGYRPRFRR
6;21-35	SAAD TKPGTTGSGAG	33;129-143	GGVP VQGSKYAADRN	60;237-251	MYRG YRPRFRRGPPR
7;25-39	TKPG TTGSGAGSGGP	34;133-147	VQGS KYAADRNHYRR	61;241-255	YRPR FRRGPPRQRQP
8;29-43	TTGS GAGSGGPGGLT	35;137-151	KYAA DRNHYRRYPRR	62;245-259	FRRG PPRQRQPREDG
9;33-47	GAGS GGPGGLTSAAP	36;141-155	DRNH YRRYPRRRGPP	63;249-263	PPRQ RQPREDGNEED
10;37-51	GGPG GLTSAAPAGGD	37;145-159	YRRY PRRRGPPRNYQ	64;253-267	RQPR EDGNEEDKENQ
11;41-55	GLTS AAPAGGDKKVI	38;149-163	PRRR GPPRNYQQNYQ	65;257-271	EDGN EEDKENQGDET
12;45-59	AAPA GGDKKVIATKV	39;153-167	GPPR NYQQNYQNSES	66;261-275	EEDK ENQGDETQGQQ
13;49-63	GGDK KVIATKVLGTV	40;157-171	NYQQ NYQNSESGEKN	67;265-279	ENQG DETQGQQPPQR
14;53-67	KVIA TKVLGTVKWFN	41;161-175	NYQN SESGEKNEGSE	68;269-283	DETQ GQQPPQRRYRR
15;57-71	TKVL GTVKWFNVRNG	42;165-179	SESG EKNEGSESAPE	69;273-287	GQQP PQRRYRRNFNY
16;61-75	GTVK WFNVRNGYGFI	43;169-183	EKNE GSESAPEGQAQ	70;277-291	PQRR YRRNFNYRRRR
17;65-79	WFNV RNGYGFINRND	44;173-187	GSES APEGQAQQRRP	71;281-295	YRRN FNYRRRRPENP
18;69-83	RNGY GFINRNDTKED	45;177-191	APEG QAQQRRPYRRR	72;285-299	FNYR RRRPENPKPQD
19;73-87	GFIN RNDTKEDVFVH	46;181-195	QAQQ RRPYRRRRFPP	73;289-303	RRRP ENPKPQDGKET
20;77-91	RNDT KEDVFVHQTAI	47;185-199	RRPY RRRRFPPYYMR	74;293-307	ENPK PQDGKETKAAD
21;81-95	KEDV FVHQTAIKKNN	48;189-203	RRRR FPPYYMRRPYG	75;297-311	PQDG KETKAADPPAE
22;85-99	FVHQ TAIKKNNPRKY	49;193-207	FPPY YMRRPYGRRPQ	76;301-315	KETK AADPPAENSSA
23;89-103	TAIK KNNPRKYLRSV	50;197-211	YMRR PYGRRPQYSNP	77;305-319	AADP PAENSSAPEAE
24;93-107	KNNP RKYLRSVGDGE	51;201-215	PYGR RPQYSNPPVQG	78;309-323	PAEN SSAPEAEQGGA
25;97-111	RKYL RSVGDGETVEF	52;205-219	RPQY SNPPVQGEVME	79;310-324	AENS SAPEAEQGGAE
26;101-115	RSVG DGETVEFDVVE	53;209-223	SNPP VQGEVMEGADN		
27;105-119	DGET VEFDVVEGEKG	54;213-227	VQGE VMEGADNQGAG		

Figure S5. Amino acids of YB-1 Peptide Array.

List and Amino acid positions of the 79 synthetic peptide fragments representing YB-1 peptide array with its consecutive 4 amino acid shift.

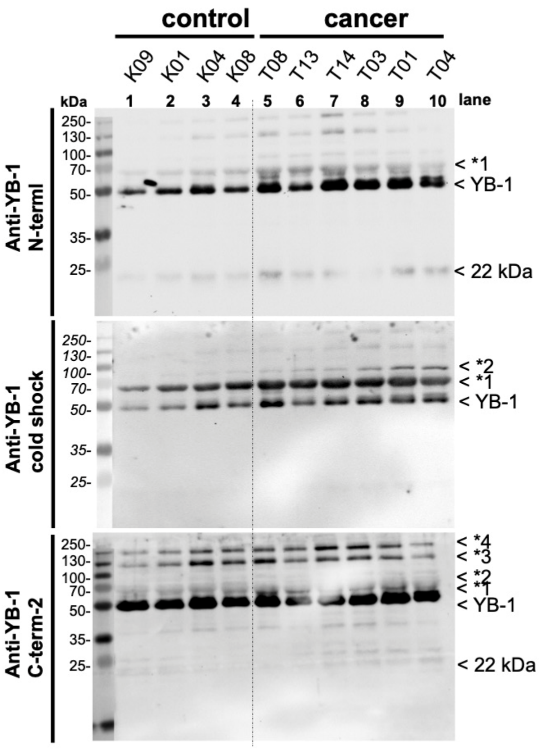

Figure S6. Immunoblotting of endogenous YB-1 in cancer and healthy controls.
Serum and plasma of healthy volunteers (lane 1-4) and cancer patients (lane 5-10) were separated on a denaturing SDS-gel, blotted on nitrocellulose and probed with N-terminal (at the top), Cold-shock domain (middle) and C-terminal (below) specific antibodies. All of the analyzed probes contain endogenous YB-1 (50 kDa). N-terminal and C-terminal domain targeting antibody also reveal a protein band of ~22 kDa. All YB-1 antibodies show protein bands of 70 kDa (*1). Cancer patients also show an additional protein band at approximately 100 kDa (*2) with cold shock domain targeting antibody. C-terminal domain targeting antibody also detect high molecular bands around 250 kDa (*3 and *4).

Table S1. Healthy controls.

Patient	Age	Sex	Tumor	Mini Mental Status Test (MMST)	CRP mg/l (ref. range: <5 ng/ml)
K01	63	w	no	30	1,2
K02	77	m	no	30	0,9
K03	74	w	no	29	1,9
K04	72	m	no	30	<0,6
K05	82	m	no	30	<0,6
K06	69	m	no	30	1,3
K07	73	w	no	29	5,5
K08	76	w	no	30	4,6
K09	75	w	no	30	1,6
K10	86	m	no	29	1,2
K11	76	w	no	29	<0,6
K12	79	m	no	30	<0,6
K13	64	w	no	30	1,1
K14	85	w	no	29	1,3

Table S2. Cancer patients.

Patient	Age	Sex	Stage	Tumor entity
T01	44	w	T3 N3 M1	Lung cancer adenocarcinoma
T02	54	w	T3 N3 M1	Lung cancer adenocarcinoma
T03	78	w		Akute myeloic leucemia
T04	42	w	IIIa	Marginal zone lymphoma
T05	60	m	T2 N2 M0	Lung cancer adenokarcinoma
T06	54	w	T3 N0 Mx	Small cell lung cancer
T07	72	m		Akute myeloic leucemia
T08	75	m	T3 N3 M1	Lung cancer squamous cell carcinoma
T09	65	w	T2 N1 M0	Gall bladder carcinoma
T10	66	m	T3 N2 M1	Lung cancer adenocarcinoma
T11	62	m	Tx N2bM1	Lung cancer squamous cell carcinoma
T12	59	w	IIa	Non-Hodgkin lymphoma
T13	72	m	T2 N3 M1	Lung cancer adenokarcinoma
T14	71	w	T2 N0M1	Breast cancer

Table S3. Densitometry readings of abbreviated GST-YB-1 protein blots.

Serum sample	Volume							
	FL	D1	D2	D3	D4	D5	D8	D9
K01	10M		18M		22M			
K03	12M	12M	24M	23M	39M	35M	84M	46M
K04	3M		3M		10M		6M	
K06	61M							15M
K07					9M			
K08	25M	16M	15M	5M	6M	7M	55M	37M
K09	7M		16M		11M	6M	7M	13M
K11	2M	3M		5M	6M		15M	14M
K13	5M	1M	7M	1M	20M	6M	1M	2M
K14	13M		26M		85M			
T01			17M	3M	14M	2M	8M	3M
T02	3M	3M	26M		11M	3M	13M	3M
T03	2M	3M	25M		22M		2M	5M
T04	13M	21M	69M	19M	32M	15M	38M	51M
T05	13M	18M	11M	10M	15M		22M	
T06	5M		18M	8M	8M	3M	11M	
T07	20M	6M	12M	8M	6M		41M	78M
T09	35M		2M	15M	31M	43M	25M	60M
T11	2M	7M	8M	7M	2M		21M	30M
T12	15M							
T13	18M	28M	22M	49M	11M		99M	109M
T14			62M	12M	45M			

Table S4. Densitometry readings of YB-1 peptide arrays.

Peptide	Serum sample											
	K01	K02	K03	K06	K09	T01	T03	T04	T06	T09	T13	T15
11			29K				86K					
12		25k	131k	85k	113k	18k	177k			77k		
13			33k	135k	53k	26k	135k			120k		
14	29k		53k	143k	124k	29k	111k			332k		
15			64k	115k	109k	19k	110k			135k		
16	28k		52k	170k			85k					
18									34k			
19				122k					50k			
21		28k	62k			17k	31k		66k			
22		46k	106k	59k	111k	53k	95k	104k	74k	484k	147k	
23		28k	125k	56k	150k	84k	134k	84k	110k	192k	209k	58k

24		51k			45k						44k
32				75k				37k			
33			76k	45k			30k	29k			
34	30k	26k	30k	74k		76k	49k	70k	40k	13k	
35	33k	73k	28k	136k	31k	64k		108k	76k	30k	
36	35k	52k	12k	66k	17k	124k		61k	28k	34k	
37	26k	54k	85k	99k	27k	162k		30k	65k	53k	
38	28k	51k	26k	113k	26k	69k			59k	34k	
45	53k	60k		87k	35k		62k				
46	33k	49k		82k	22k	75k	45k	39k		59k	
47	44k	83k	52k	111k	56k	34k	65k	36k			38k
48	39k	65k	32k	83k	46k	73k		20k	64k	54k	
49	38k	55k	51k	79k	45k	47k		27k	101k	40k	61k
50			47k						121k	55k	87k
56	25k			83k							
57	36k	45k		120k	21k	35k		37k		20k	
58	26k	56k	31k	142k	18k	41k	44k	48k	79k	28k	
59	64k	69k	42k	159k	24k	82k	41k	78k	81k	30k	
60	55k	74k	27k	125k	23k	64k	43k	82k	54k	21k	
61	36k	76k	36k	129k	18k	69k			52k	34k	
68	40k	82k	39k	145k	38k	45k	44k	34k	82k	34k	
69	26k	87k	37k	112k	40k	74k	18k	50k	101k	47k	
70	53k	111k	59k	191k	74k	134k	85k	48k	134k	62k	34k
71		61k						86k			
72								80k			
73								56k			
74								65k			

Original blots

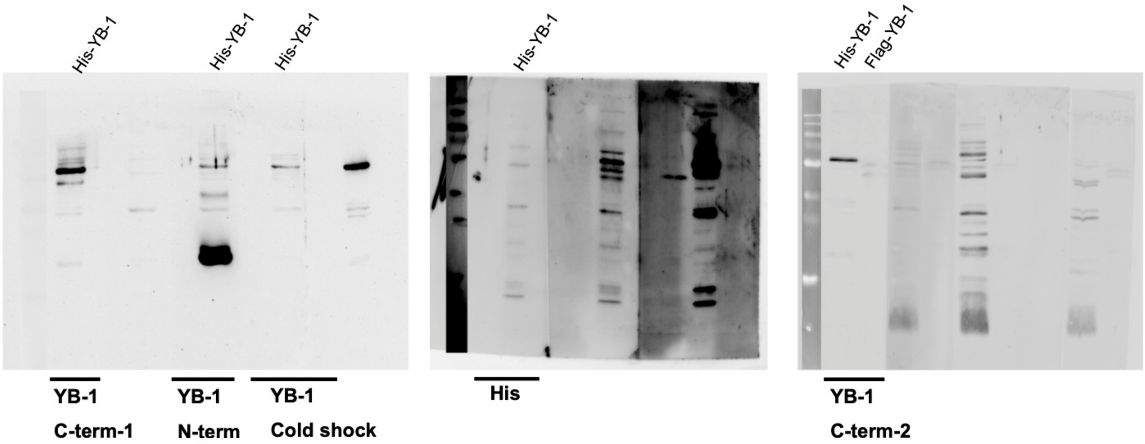

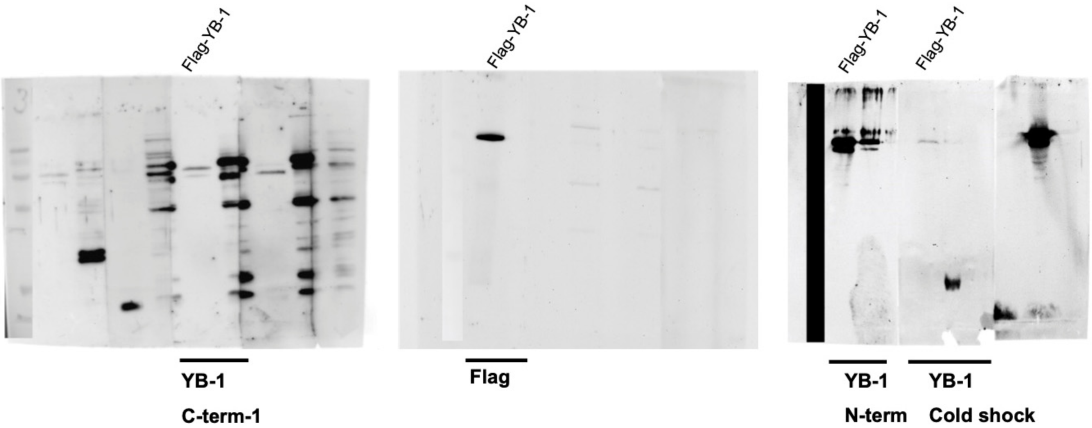

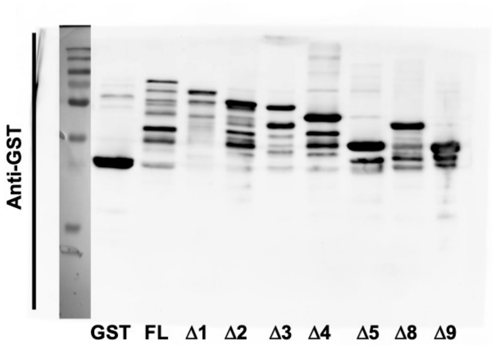

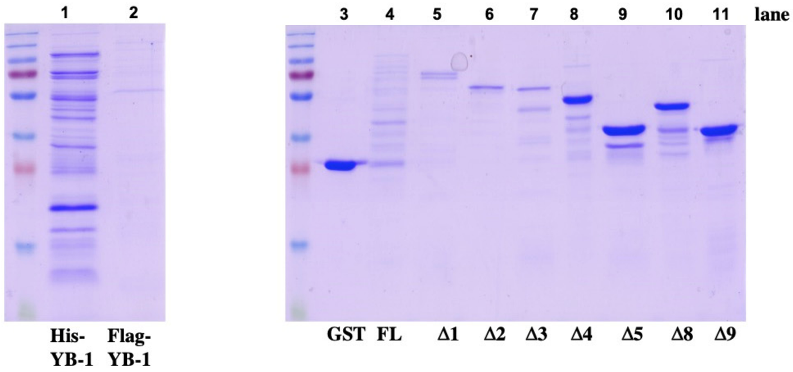

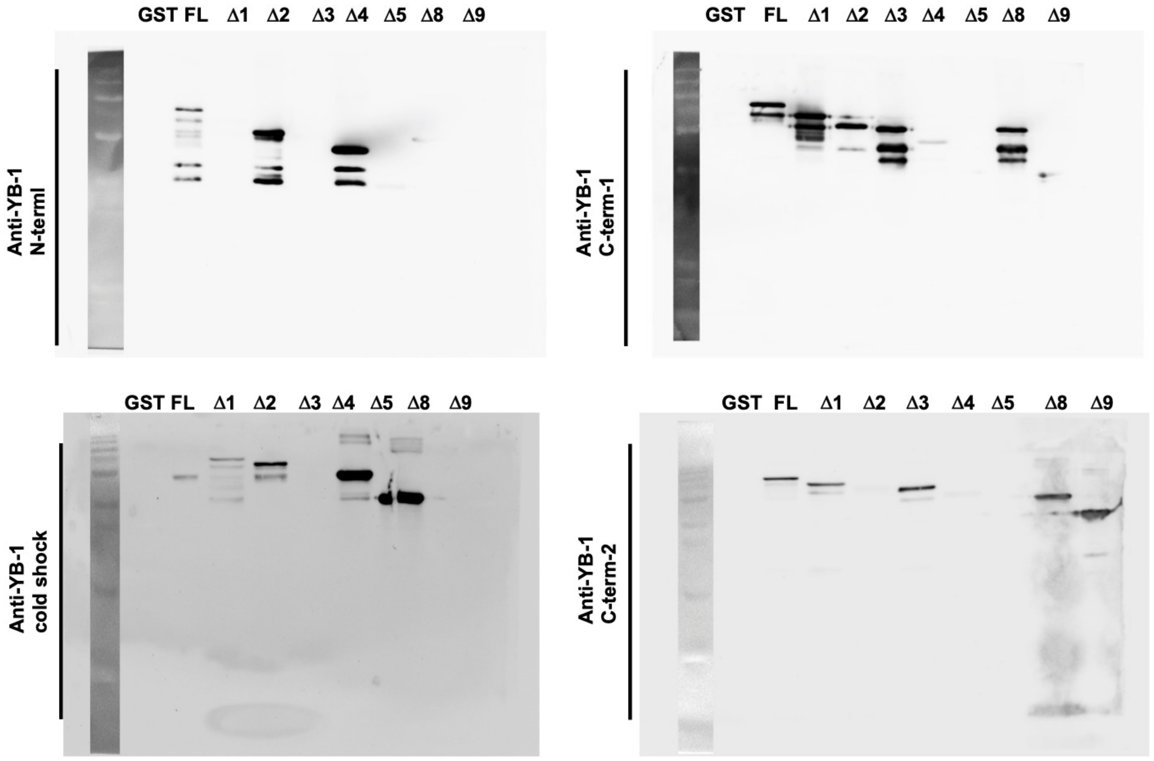
GST FL Δ1 Δ2 Δ3 Δ4 Δ5 Δ8 Δ9
Anti-YB-1 N-terml
GST FL Δ1 Δ2 Δ3 Δ4 Δ5 Δ8 Δ9
Anti-YB-1 C-term-1
GST FL Δ1 Δ2 Δ3 Δ4 Δ5 Δ8 Δ9
Anti-YB-1 cold shock
GST FL Δ1 Δ2 Δ3 Δ4 Δ5 Δ8 Δ9
Anti-YB-1 C-term-2

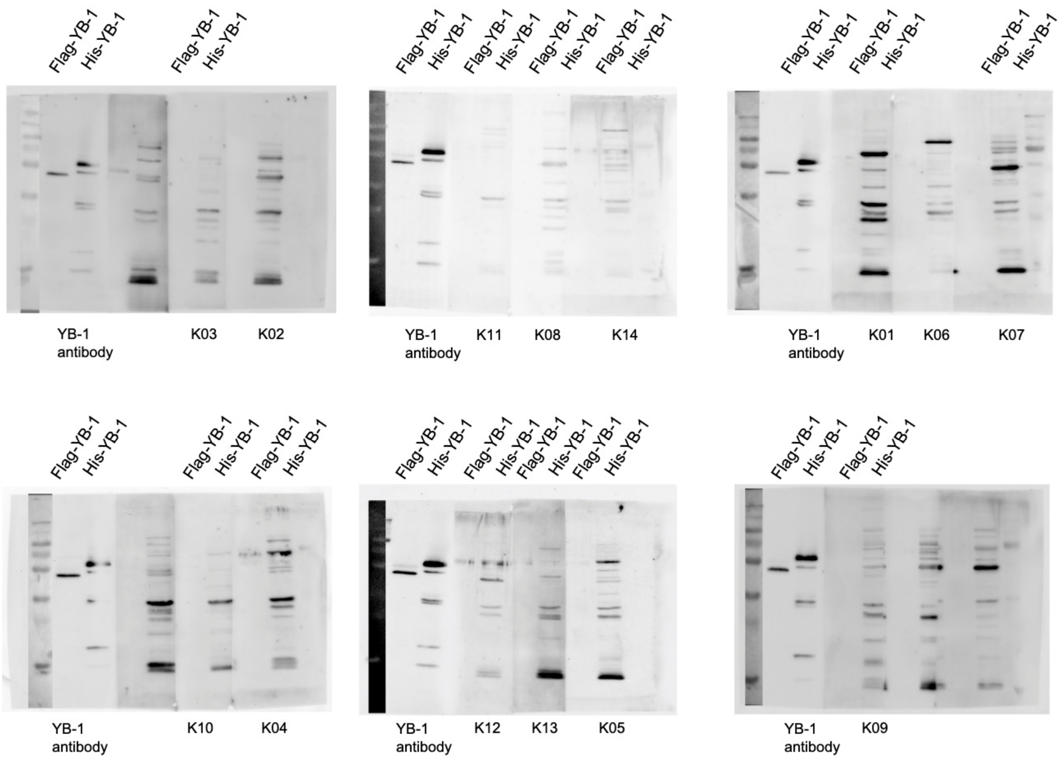
Flag-YB-1
His-YB-1
YB-1 antibody
K03
K02
YB-1 antibody
K11
K08
K14
YB-1 antibody
K01
K06
K07
YB-1 antibody
K10
K04
YB-1 antibody
K12
K13
K05
YB-1 antibody
K09

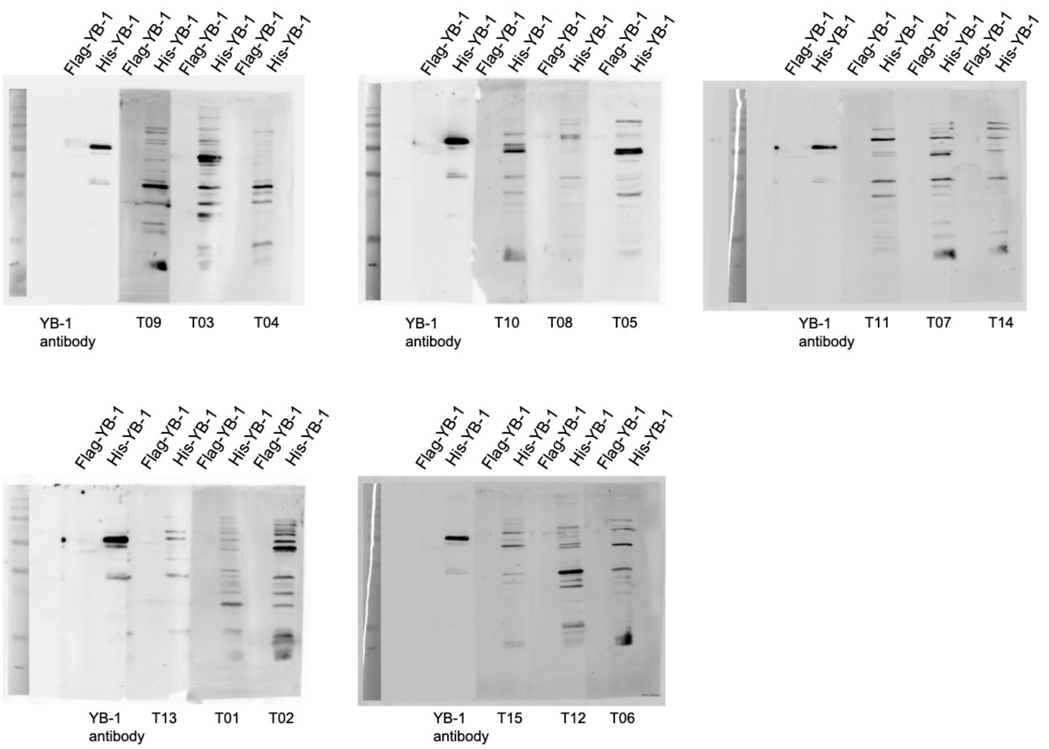
Flag-YB-1
His-YB-1
YB-1 antibody
T09
T03
T04
T10
T08
T05
T11
T07
T14
T13
T01
T02
T15
T12
T06

A **B**

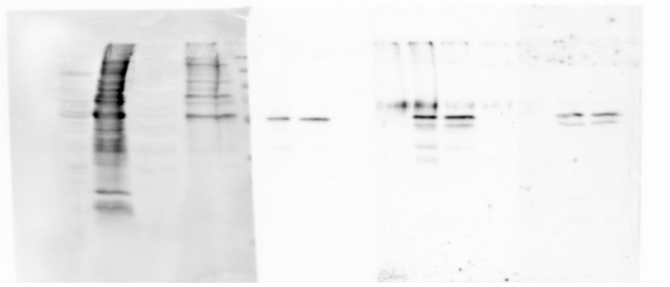

C

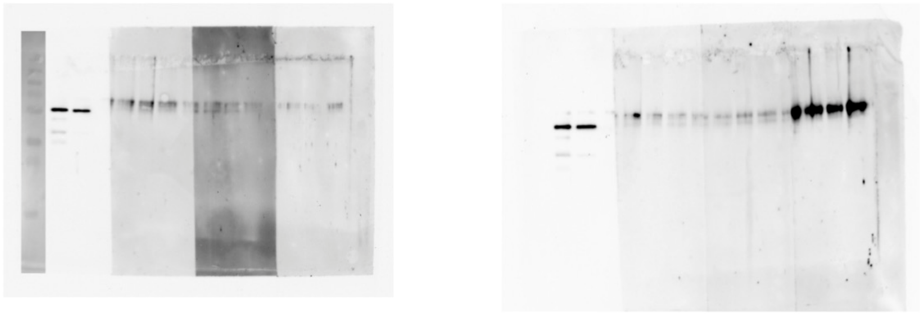

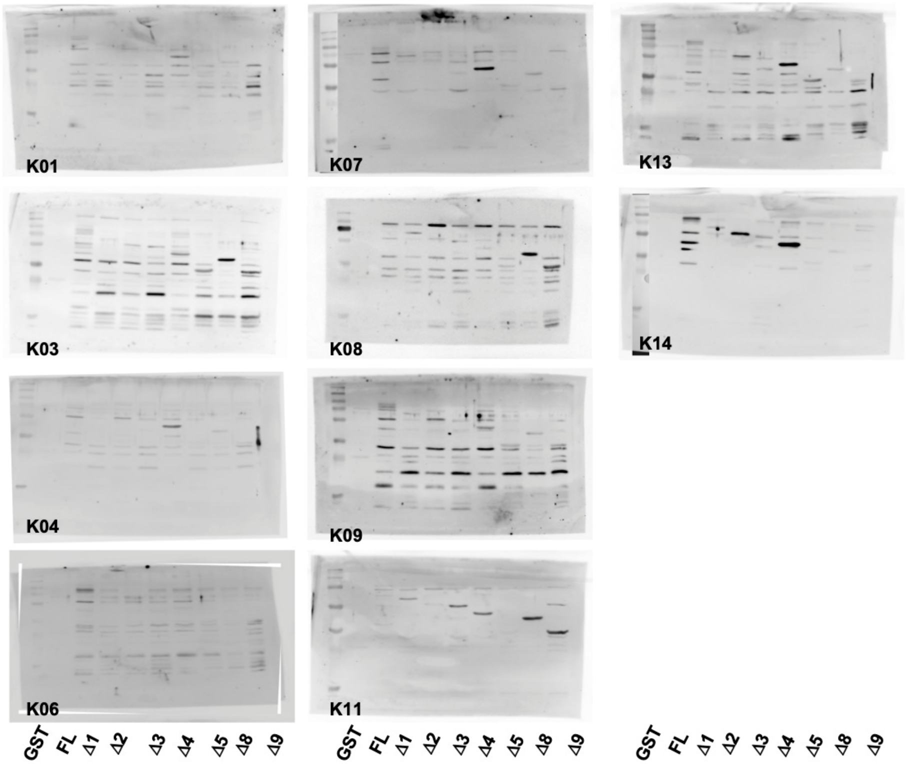
K01
K07
K13
K03
K08
K14
K04
K09
K06
K11
GST FL Δ1 Δ2 Δ3 Δ4 Δ5 Δ8 Δ9
GST FL Δ1 Δ2 Δ3 Δ4 Δ5 Δ8 Δ9
GST FL Δ1 Δ2 Δ3 Δ4 Δ5 Δ8 Δ9

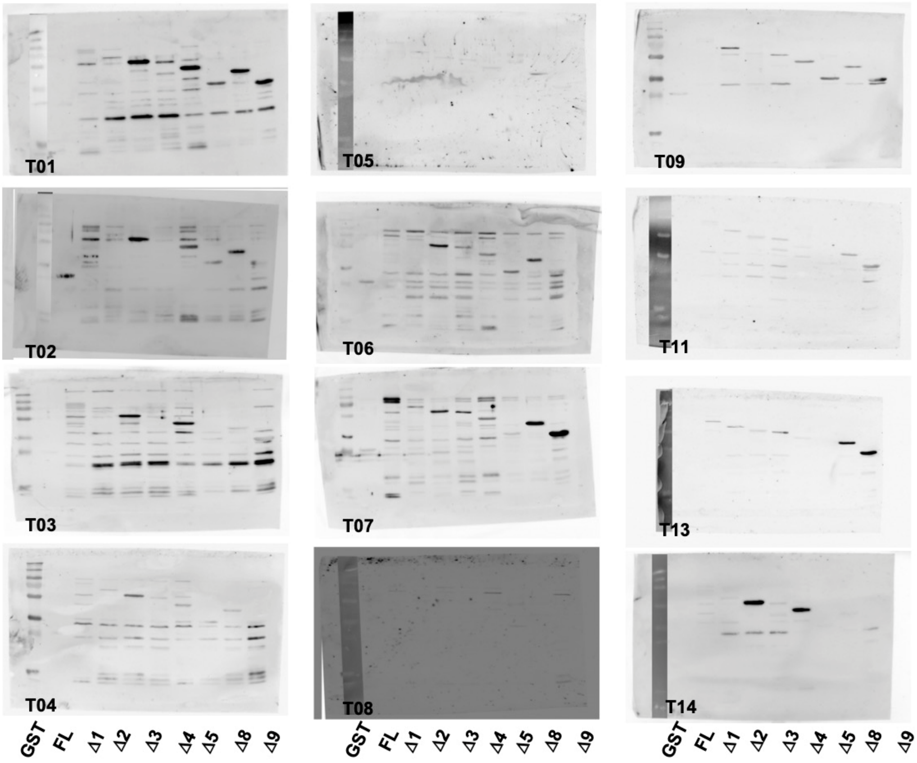
T01
T05
T09
T02
T06
T11
T03
T07
T13
T04
T08
T14
GST FL Δ1 Δ2 Δ3 Δ4 Δ5 Δ8 Δ9
GST FL Δ1 Δ2 Δ3 Δ4 Δ5 Δ8 Δ9
GST FL Δ1 Δ2 Δ3 Δ4 Δ5 Δ8 Δ9

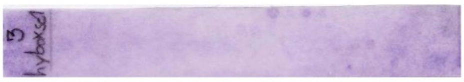
K01
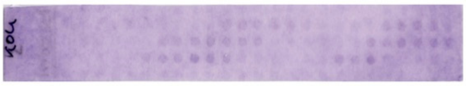
K02
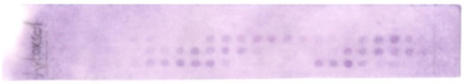
K03
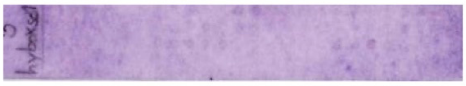
K04
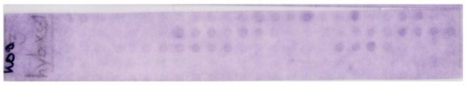
K06
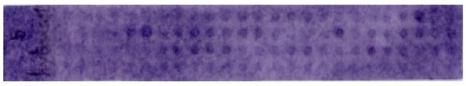
K08
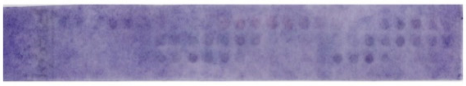
K09
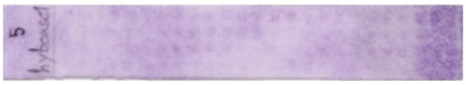
K10
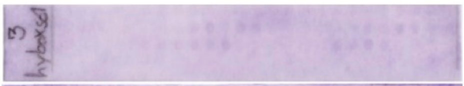
K11
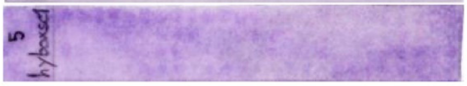
K12
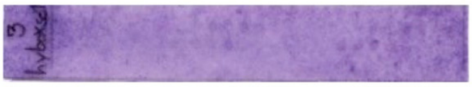
K13
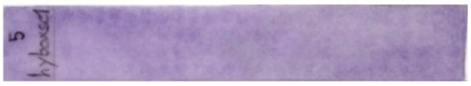
K14

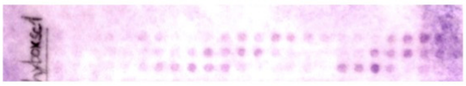
T01
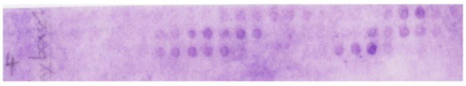
T03
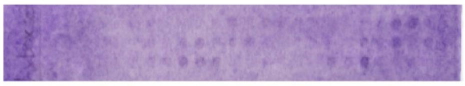
T04
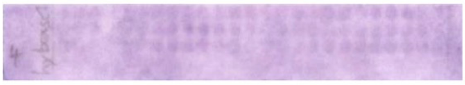
T06
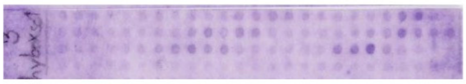
T09
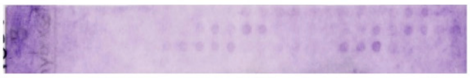
T13
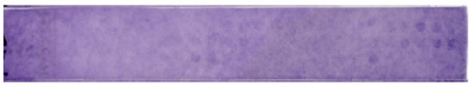
T15

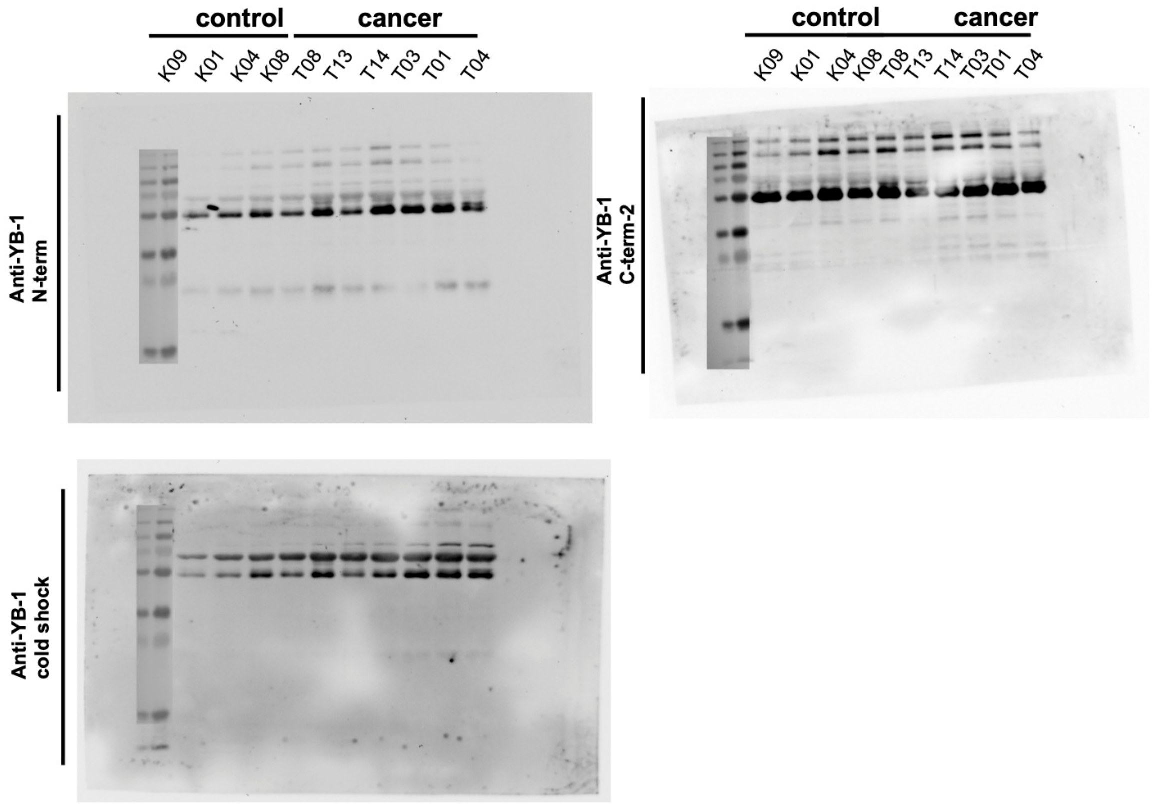
control
cancer
K09
K01
K04
K08
T08
T13
T14
T03
T01
T04
Anti-YB-1
N-term
Anti-YB-1
C-term-2
Anti-YB-1
cold shock

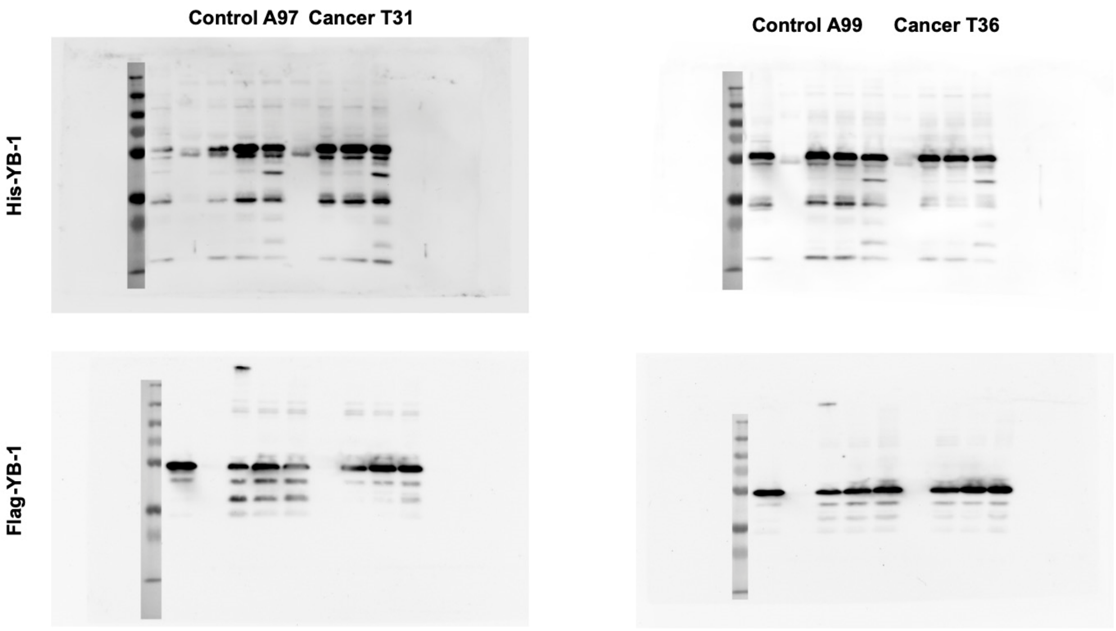
Control A97 Cancer T31
Control A99 Cancer T36
His-YB-1
Flag-YB-1

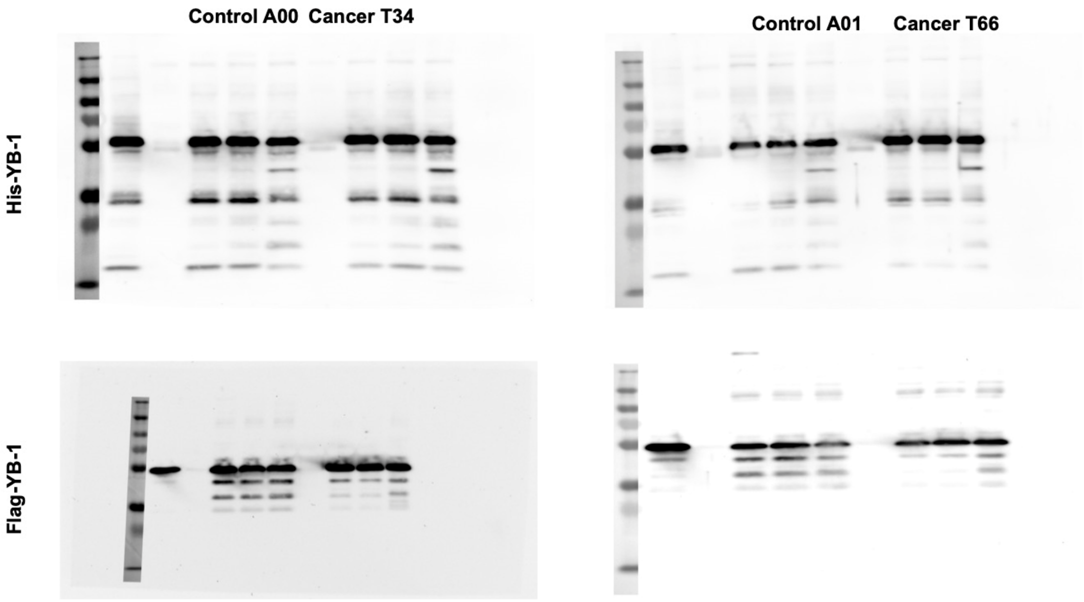
Control A00 Cancer T34
Control A01 Cancer T66
His-YB-1
Flag-YB-1
